# Vericiguat reduces atrial fibrillation recurrence by alleviating myocardial fibrosis via the TGF-β1/Smad2/3 pathway

**DOI:** 10.1371/journal.pone.0328272

**Published:** 2025-07-18

**Authors:** XiaoLin Sun, Yawen Sheng, Pei Xu, Qian Peng, Zhongbao Ruan

**Affiliations:** 1 Department of Cardiology, The Affiliated Taizhou People’s Hospital of Nanjing Medical University, Taizhou, Jiangsu, The People’s Republic of China; 2 Department of Hematology, The Affiliated Taizhou People’s Hospital of Nanjing Medical University, Taizhou, Jiangsu, The People’s Republic of China; 3 Nanjing University of Chinese Medicine, Nanjing, Jiangsu, The People’s Republic of China; Nanjing First Hospital, Nanjing Medical University, CHINA

## Abstract

Atrial fibrillation (AF) and heart failure (HF) are mutually reinforcing, and the prognosis for both diseases is poor. Vericiguat is the first oral soluble guanylate cyclase (sGC) stimulator to be approved for the treatment of symptomatic, ejection fraction-reduced chronic heart failure (HFrEF) in adults. It exerts a general therapeutic effect on cardiovascular diseases, with a particular efficacy in the treatment of HF. However, it remains uncertain whether vericiguat exerts a therapeutic effect on atrial fibrillation. The objective of this study was to investigate the potential mechanism of vericiguat in the treatment of atrial fibrillation. A retrospective analysis was conducted to investigate the effects of vericiguat on patients with heart failure and paroxysmal AF. Furthermore, the effects of vericiguat on AF and the degree of myocardial fibrosis in rat AF models and cells were observed. It was found that vericiguat may control the recurrenceof AF in clinical studies and can control the fibrosis of AF rats in vivo and in vitro experiments. RNA-Seq sequencing revealed that the TGF-β1/Smad pathway in cells treated with vericiguat was significantly enriched. In vitro validation demonstrated that the anti-fibrotic effect of Vericiguat was weakened by the TGF-β1/Smad pathway when Protein Kinase G (PKG) was knocked down. The findings indicate that vericiguat may inhibit myocardial fibroblast activation and collagen synthesis via the TGF-β1/Smad pathway, thereby exerting a controlling effect on the recurrence of atrial fibrillation.

## Introduction

Atrial fibrillation (AF) is a recurrent arrhythmia commonly found in adults over the age of 20, with a prevalence rate of approximately 3% [1]. It is a cardiovascular disease with a high incidence rate, high mortality rate and high medical costs. The risk of stroke in patients with atrial fibrillation is fivefold that of individuals without the condition. Moreover, patients with atrial fibrillation may experience atrial enlargement, which can ultimately lead to heart failure (HF) and significantly impact their quality of life. The primary research focus in the field of atrial fibrillation pathogenesis is structural remodeling. Structural remodeling is characterized by three main features: increased atrial volume, ultrastructural changes in myocardial cells, and atrial muscle fibrosis [2,3]. The degree of atrial tissue fibrosis is the most significant feature of atrial remodeling [4,5]. Fibrosis causes extracellular matrix (ECM) deposition surrounding myocardial cells, changes in fibroblast proliferation and phenotypes that lead to alterations in ECM synthesis and degradation. The interactions between structural restructuring and electrical remodeling are believed to be responsible for the triggering and maintenance of AF [6]. HF is a common manifestation of cardiac dysfunction. It is caused by long-term ventricular overload, which leads to cardiac circulatory dysfunction [7–9]. The population at greatest risk of developing HF is the elderly. Once the condition has developed, it is difficult to recover. If it further develops, it can lead to complications such as AF and arrhythmia. HF complicated by AF can lead to a decrease in cardiac ejection function, further exacerbating the condition of HF [3,10]. The precipitating factors for HF and AF are partially analogous, thereby rendering these two diseases interactive. Consequently, there is an urgent necessity to identify a pharmaceutical agent that can treat both HF and AF.

Vericiguat is a novel anti-heart failure drug that is an oral soluble guanosine monophosphate (sGC). It enhances the cyclic guanosine monophosphate (cGMP) pathway by directly stimulating soluble guanosine cyclase, without relying on nitric oxide. In addition, vericiguat enhances the sensitivity of soluble guanylate cyclase to endogenous nitric oxide by stabilizing the nitric oxide binding site [11,12]. Previous studies have demonstrated that the NO-sGC-cGMP axis represents a pivotal signaling pathway regulating the cardiovascular system. Additionally, promoting cGMP has been shown to reduce vascular smooth muscle migration by inhibiting the TGF-β1/Smad signaling pathway [13,14]. While, the TGF-β1/Smad signaling pathway represents the most significant pathway in the context of fibrosis. Additional research has demonstrated that sGC agonists can attenuate glomerulosclerosis, renal and hepatic fibrosis, and skin fibrosis to varying degrees [15]. However, it remains uncertain whether vericiguat exerts a therapeutic effect on atrial fibrillation. The objective was to ascertain whether vericiguat could mitigate myocardial fibrosis in patients with AF, thereby reducing the likelihood of AF recurrence and elucidating the underlying mechanism.

## Materials and methods

### Patient selection,Vericiguat therapy and collected data

This retrospective study included patients who received vericiguat between 01/09/2021 and 01/11/2022. In the event that patients with HFrEF and paroxysmal AF experience a deterioration in their symptoms of HF subsequent to the administration of guideline-guided medication, the attending cardiologist will determine whether to use vericiguat treatment. The dose titration of vericiguat was conducted in accordance with the manufacturer-recommended regimen. Vericiguat was initiated at a dosage of 2.5 mg and subsequently increased to 10 mg. In the event that patients exhibited systolic blood pressure below 90 mmHg or presented with hypotensive symptoms, including dizziness and lightheadedness, the dose up-titration was postponed.

Baseline characteristics, including laboratory, echocardiographic, and medication data, were collected for all subjects. Several laboratory data, were also obtained 6 (±2) months after the initiation of vericiguat. This study was conducted in accordance with the “Declaration of Helsinki” and was previously approved by the local institutional review board (number KY 2022-018-01). The requirement for written informed consent was waived due to the retrospective nature of the study and the opt-out methodology employed.

### Animals

A total of 20 healthy Wistar rats (male, 220–250 g) were obtained from Yangzhou University and were randomly assigned to three individual experiments, with three rats being used in each experiment. The rats were provided with standard care in accordance with a 12-hour dark/light cycle (25°C with an atmosphere of 60% relative humidity) and were permitted free access to food and water. Prior to the commencement of the experiment, the rats were subjected to a seven-day period of adaptive feeding. The health and behavior of the animals were observed on a daily basis until death. All procedures involving the care and use of animals were conducted in accordance with the guidelines set forth by the Animal Research: Reporting of In Vivo Experiments (ARRIVE) initiative and were approved by the animal care guidelines of the Laboratory Animal Management and Experimental Animal Ethics Committee of Yangzhou University (approval no. 202303545). All surgery was performed under sodium pentobarbital anesthesia, and all efforts were made to minimize suffering. All rats were anesthetized with 20% sodium urethane (0.2 g/ml; 1.0 g/kg intraperitoneal injection) and subsequently sacrificed by cervical dislocation. Animal mortality was verified by ascertaining cardiac and respiratory arrest.

### Rat model of AF and fibrosis assessment

Rats were divided into three groups: the control group (blank group), AF group and vericiguat group (AF group treatment with vericiguat). The rat model of AF was induced by Ach (66 μg/mL; Solarbio Science & Technology Co., Ltd)-CaCl_2_ (50 mg/mL; Solarbio Science & Technology Co., Ltd) at a dose of 0.1 mL/100 g via tail vein injection for 28 days [16]. Two criteria were used to evaluate the successful establishment of the AF model: 1) changes in the electrocardiogram and 2) assessment of pathological fibrosis of the left atrium. Four days after the establishment of the AF model, vericiguat (0.5 mg/kg) was administered once a day via oral gavage for a period of eight weeks [17]. At the end of the study, all the rats were euthanized and their hearts were promptly collected for further study.

### Isolation and culture of cardiac fibroblasts in neonatal rats

Rat cardiac fibroblasts (CFs) were isolated from neonatal rats aged between one and three days old. The hearts were excised from the rats and divided into approximately 1 mm³ pieces. The tissues were digested with trypsin and type II collagenase. The cells were pelleted by centrifugation at 1000 rpm for 5 min. The cell pellets were resuspended in Dulbecco’s modified Eagle’s medium with 10% fetal bovine serum (FBS; Gibco), and 1% penicillin/streptomycin, plated onto a T25 flask, and incubated 37°C for 2 h. CFs were collected after differential adherence. Following the incubation period, the media was replaced with complete media, and the cells were maintained at 37°C in a humidified atmosphere containing 5% CO₂ for the duration of the experiment. CFs of the second or third passage were used in our experiments.

### Cell migration, viability, and proliferation assays

To determine the migration capacity of CFs using wound healing methods, we divided CFS into three groups. The control group was not subjected to any intervention, whereas the AngII group was treated with AngII for a period of 24 hours. The vericiguat + AngII treatment group was treated with AngII for 24 hours, after which vericiguat was administered for a further 24 hours. Inhibitor group was treated with AngII and 1umol·L-1KT5823 for 24 hours, The cells were seeded in a 6-well plate and allowed to culture overnight until they reached confluence. The cell monolayers were incised using sterile pipette tips with a 200 μL capacity, and the detached cells were removed by washing with phosphate-buffered saline. The cells were then incubated with the indicated treatments, and the wounds were imaged at 0 and 24 h using an inverted microscope (BX53F2, Olympus, Tokyo).

### Transcriptome resequencing (RNA-seq)

To explore the specific treatment mechanism of vericiguat on AF, particularly with regard to structural remodeling, we performed RNA-Seq of CFs in each experimental group (blank group, the AngII group, and the vericiguat + AngII treatment group). Bioinformatics was used to analyze and screen the possible mechanism of vericiguat treatment on myocardial fibrosis. The raw sequencing data are termed raw reads. First, the reads exhibiting low quality, joint contamination, and a high N content of an unknown base were filtered out. The filtered data were termed clean reads. The clean reads were then compared with the reference genome, and new transcript prediction, single-nucleotide polymorphism (SNP) detection, insertion-deletion (INDEL), and differential gene splicing were performed. After obtaining new transcripts, new transcripts with protein coding potential were added to the reference gene sequence to form a complete reference sequence, and the level of gene expression was calculated. Finally, for multiple samples, the differentially expressed genes (DEGs) between the different samples were detected as needed. A comprehensive cluster and functional enrichment analysis was conducted on the DEGs.

### Immunofluorescence

The growth of adherent cells was observed after 24 h through the use of immunofluorescence staining. CFs were fixed with 4% paraformaldehyde at 4°C for 30 min. Fixed cells were permeabilized with 0.1% Triton X-100 and blocked with blocking solution (2% FBS/0.1% Tween 20). Fixed cells were incubated overnight at 4°C with primary antibodies (1:200 dilution) against rat α-SMA (ab7817; Abcam plc). Cells stained with diluent only served as the negative control. After overnight incubation, cells were washed with PBS three times for 5 min each, incubated with CY3-conjugated secondary antibody (1:500; ab6939; Abcam plc) for 1 h at room temperature, and washed five times with PBS. Nuclei were stained with 4′,6-diamidino-2-phenylindole. Slides were mounted and examined using a fluorescent microscope (TE2000, Nikon, Tokyo).

### Real-time quantitative polymerase chain reaction (qRT-PCR)

Total RNA was extracted by TRIzol1 (DP424, TIANGEN) and cDNA was synthesised from sample RNA using the FastKing RT Kit (KR116, TIANGEN) following the manufacturer’s instructions. cDNA was amplified by PCR through the use of a Qiagen PCR kit according to the manufacturer’s instructions at 50°C for 30 min, 95°C for 3 min, 95°C for 15 sec and 60°C for 30 sec. α-SMA, Collagen I, Smad2, Smad3, Collagen III, TGF-β1 and Fibronectin were detected by qRT‑PCR. The 20 µl PCR amplification reaction included 2 µl cDNA, 10 µl SYBR Taq, 0.8 µl forward primer, 0.8 µl reverse primer, 0.4 µl RoxII and 6 µl double‑distilled water. Subsequently, PCR reaction was achieved on the basis of the two‑step procedure (95°C for 15 min; 95°C for 10 sec and 60°C for 32 sec) and procedure was repeated 40 times. Primers applied were shown in S1 Tables. The PCR instrument for qRT‑PCR was ABIPRISM 7500. Each experimental condition was conducted in triplicate. The relative mRNA quantities were determined using the 2^-ΔΔCq^ method.

### Western blotting

Proteins were extracted from the cells using RIPA lysis buffer containing 1%PMSF (C1055, Pulilai BioTech) to prevent protein degradation. Protein concentrations were measured using the BCA method (CW0014, CWBIO). The protein samples (10 μg/lane) were then separated using 10% SDS-PAGE and electrophoresed. After electrophoresis, the proteins were transferred to PVDF membranes. Subsequently, the membranes were blocked for 2 h at room temperature by incubation in TBST containing 5% nonfat milk. Primary antibodies against α-SMA (1:1000; ab7817; Abcam plc), Collagen I (1:1000; ab270993; Abcam plc), Smad2 (1:1000; ab280888; Abcam plc), Smad3 (1:1000; ab208182; Abcam plc), Collagen III (1:1000; ab84993; Abcam plc), TGF-β1 (1:1000; ab215715; Abcam plc), β-actin (1:1000; ab8226; Abcam plc) and Fibronectin (1:1000; ab268020; Abcam plc) were incubated overnight at 4°C. Finally, the membranes were incubated with corresponding secondary antibodies for 2 h at room temperature and detected by an ECL Chemiluminescent Substrate Reagent Kit (Thermo Fisher Scientific, Inc.).

### Histopathology

Rats heart tissues were fixed in 4% paraformaldehyde, embedded in paraffin, and sectioned at a thickness of 5 µm. H&E staining was performed to observe pathological alterations in the heart. Masson’s trichrome staining was used to examine changes in collagen deposition in left ventricular tissues. Immunohistochemistry was performed using α-SMA antibody. Random fields were selected from each section and observed under the microscope. One image of each section was acquired and analyzed by Image-Pro Plus software.

### Statistical analysis

All data were analyzed using the Prism 8.0 (GraphPad). The results are presented as the mean ± standard deviation. Statistical significance was assessed with Student’s t test or analysis of variance (ANOVA) with Tukeys post hoc test. A p-value <0.05 was considered to be statistically significant.

## Results

### Vericiguat can effectively improve cardiac function and reduce the recurrence of AF in patients with AF complicated with HF

**Fig 1:**
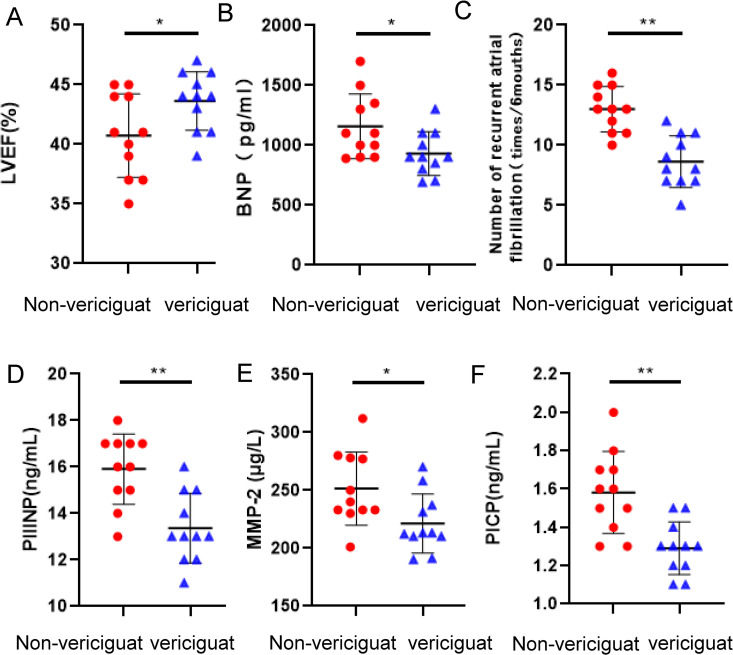
Effects of Vericiguat Treatment on patients with atrial fibrillation and heart failure. (A) LVEF, (B) Serum BNP, (C) the recurrence rate of AF (D) PIIINP, (E) MMP-2, and (F) PICP levels were shown in 22 patients at 6 months with or without vericiguat anti-heart failure therapy. * p < 0.05 and **p < 0.01.

In accordance with the established inclusion criteria, 22 patients diagnosed with paroxysmal AF with HF were included in this study. Patients were divided into two groups based on the presence or absence of vericiguat treatment: the vericiguat treatment group and the control group, with 11 cases in each group. Both groups received standardized anti-heart failure treatment. There was no statistically significant difference in the duration of paroxysmal AF, LVEF, heart rate, and medication use between the two groups (Table 1, p > 0.05). Following treatment, the therapeutic effects of the two groups were compared after 6 months. There was no significant difference in the hospitalization rate between the two groups (Table 2, p > 0.05), but HF indicators such as BNP and LVEF values were significantly improved in vericiguat treatment group (Fig 1A and 1B, p < 0.05). Notably, the recurrence rate of AF was reduced in the vericiguat group (Fig 1C, p < 0.05). Further research revealed that the myocardial fibrosis indicators, including PIIINP, MMP-2, and PICP, were significantly reduced in the vericiguat group (Fig 1D-F, p < 0.05). The two groups exhibited comparable all-cause hospitalization and all-cause mortality rates. The results demonstrated that vericiguat not only improved HF but also effectively controlled the recurrence of AF and improved myocardial fibrosis.

**Table 1:** Demographic and clinical characteristics of the patients at baseline.

Variable	Non- vericiguat (n = 11)	vericiguat (n = 11)
**Demographics**		
Age (years)	67.45 ± 11.79	64.55 ± 9.585
Male gender(%)	6(54.55)	7 (63.64)
BMI(kg/m2)	23.27 ± 1.679	23 ± 1.483
**History**		
Hypertension(%)	5/11(45.45)	5/11(45.45)
Diabetes mellitus(%)	2/11 (18.18)	3/11 (27.27)
Hypercholesterolemia(%)	3/11 (27.27)	4/11 (36.36)
History of MI(%)	1/11 (9.09)	2/11 (18.18)
Stroke(%)	2/11(18.18)	1/11 (9.09)
**Data on hospital admission**		
LA(mm)	45.45 ± 2.296	45.36 ± 2.335
LVEF(%)	39.36 ± 3.931	39.55 ± 3.725
BNP (Pg/ml)	1315 ± 344.4	1388 ± 375.1
**Myocardial fibrosis index**		
PIIINP(ng/mL)	16.27 ± 1.618	17 ± 2.366
MMP-2 (μg/L)	266.8 ± 23.6	258.6 ± 25.57
PⅠCP(ng/mL)	1.6 ± 0.2408	1.555 ± 0.2067
**Medication**		
statin(%)	4/11(36.36)	5/11(45.45)
Sacubitril/valsartan(%)	11/11(100)	11/11(100)
Beta blocker(%)	11/11(100)	11/11(100)
Diuretics(%)	11/11(100)	11/11(100)
anticoagulant drugs (%)	11/11(100)	11/11(100)
SGLT2 inhibitor(%)	9/11(81.82)	10/11(91.67)

BMI, body mass index; MI, myocardial infarction; LA, left atrial; LVEF, left ventricular ejection fraction.

**Table 2:** Study end points at 6 months.

	Non- vericiguat(n = 11)	Vericiguat(n = 11)
Comorbidity (%)		
History of stroke or TIA (%)	0(0.00)	0(0.00)
AHF (%)	2(18.1)	1(9.09)
•Cardiac death (%)	0(0.00)	0(0.00)
All-cause	1.455 ± 0.9342	0.9091 ± 0.7006
Hospitalizations	1 ± 0.2	0.8 ± 0.3

TIA, Transient Ischemic Attack; AHF, acute heart failure.

### The in vitro confirmation of the anti-fibrotic effect of vericiguat on myocardial cells

**Fig 2:**
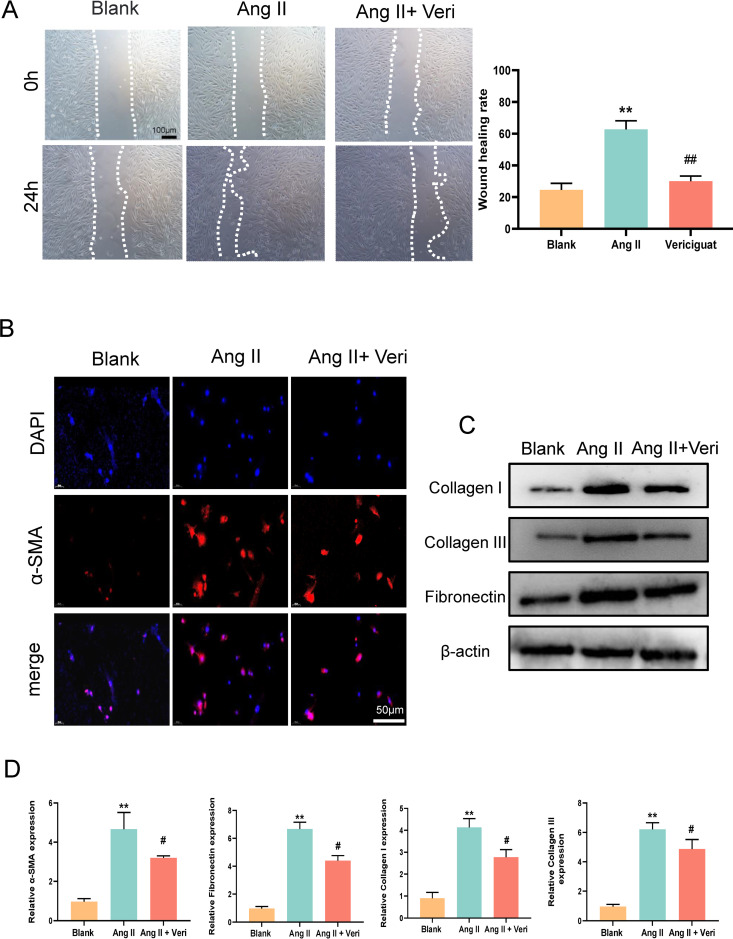
In vitro validation of the antifibrotic effect of vericiguat on cardiomyocytes. (A) Wound healing assay to detect the migratory capacity of CMs after Ang Ⅱ and vericiguat treatments. The left panel shows images of the wound area at 0h and 24h after scratching; the right panel shows the quantitative analysis of the migration rate. (B) Immunofluorescence analysis was used to evaluate the level of α-SMA in CMs after different treatments. Scale bar, 100 μm. (C) Detection of myocardial fibrosis of key proteins expression under different treatments in CMs. (D) qRT-PCR detection of mRNA changes in key genes of the myocardial fibrosis (^*^p < 0.05 versus Blank group, ^**^ p < 0.01 versus Blank group, ^#^p < 0.05 versus vericiguat group, ^##^p < 0.01 versus vericiguat group), Veri, vericiguat.

To investigate the role of vericiguat in anti-fibrosis, we utilized Ang II to induced CFs into myofibroblasts. Subsequently, the effects of vericiguat treatment on CFs were evaluated, including their proliferation, migration, and collagen synthesis abilities. The present study revealed that, in comparison with the control group, the myocardial cGMP and PKG levels in the AngII model group were significantly reduced. In comparison with the AngII model group, the levels of myocardial cGMP and PKG protein in the AngII+ vericiguat group were significantly increased (S1 Fig). The scratch healing experiment demonstrated that treatment with Ang II facilitated the migration of CFs, which is consistent with previous studies [20], while vericiguat reversed the effect of Ang II (Fig 2A). In order to further investigate the effect of vericiguat on CFs, we evaluated the expression of myocardial fibrosis-specific genes (α-SMA, collagen I, and collagen III) in CFs using qRT-PCR and Western blot analysis. Research has demonstrated that the action of Ang II results in the upregulation of the visceral-specific contractile proteins α-SMA and collagen I in cardiac fibroblasts, accompanied by an increase in fibronectin expression. Immunofluorescence analysis revealed an increase in α-SMA expression in CFs treated with Ang II (Fig 2B). In comparison to the Ang II group, the expression of fibronectin was found to be reduced following treatment with vericiguat. Similarly, the expression of α-SMA and collagen I in CFs treated with vericiguat was diminished, and the levels were comparable to those of blank CFs. The aforementioned outcomes substantiate that vericiguat is efficacious in safeguarding CMs from oxidative stress-induced damage, reducing the expression of fibronectin, and facilitating heart repair (Fig 2C and 2D).

### TGF-β1/smad pathway plays a role in the process of myocardial fibrosis

**Fig 3:**
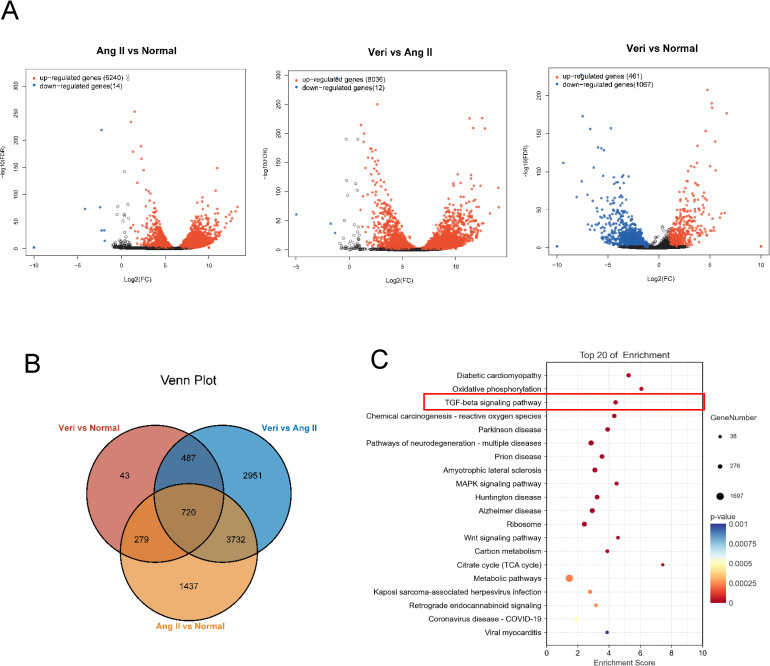
Transcriptome sequencing analyzes differential genes between Ang II group, vericiguat and blank group. (A) Volcano plot of differential gene expression in Ang II vs blank, vericiguat vs Ang II and vericiguat vs blank group. (B) Venn plot for the three sets of common differentially expressed genes. (C) KEGG analysis of enrichment pathways for differentially expressed genes. Veri, vericiguat.

In order to elucidate the molecular mechanism of reducing myocardial fibrosis and treating AF with vericiguat, transcriptome sequencing was conducted on CFs derived from the blank group, the AngII group, and the vericiguat + AngII treatment group, respectively. The RNA-Seq data from this study have been submitted and archived to the NCBI Sequence Read Archive under sample accession number GSE278356. Gene expression analysis revealed that 6254, 8048, and 1528 differentially expressed genes were identified between each group (Fig 3A, | log2FC | ≥ 1 and p < 0.05). Following Venn analysis, 720 common differentially expressed genes were identified (Fig 3B). Further KEGG analysis revealed that these genes were highly enriched in the TGF-β1 pathway (Fig 3C), indicating a high correlation between the TGF-β1 pathway and the pathogenesis of AF and the therapeutic effect of vericiguat. This discovery is also consistent with the important role of this pathway in the pathogenesis of AF [18,19].

### Vericiguat inhibits TGF β/Smad2/3 pathway through activated PKG

**Fig 4:**
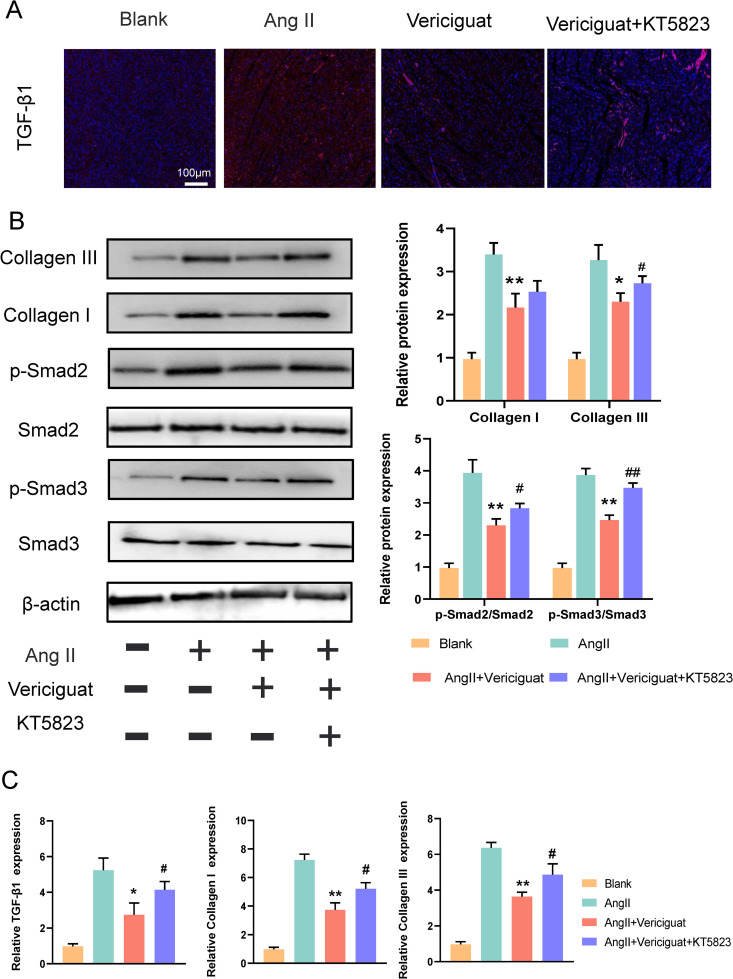
Vericiguat regulates TGF- β1/Smad2/3 pathway through PKG. (A) Representative immunofluorescence staining for TGF-β1 in different treatment groups. (B) Detection of TGFβ1/Smad2/3 pathway and myocardial fibrosis of key proteins expression under different treatments in rat left atrial tissue. (C) qRT-PCR detection of mRNA changes in key genes of the TGF-β1/Smad2/3 pathway and myocardial fibrosis (^*^p < 0.05 versus Ang II group, ^**^p < 0.01 versus Ang II group, ^#^p < 0.05 versus vericiguat group, ^##^p < 0.01 versus vericiguat group).

**Fig 5:**
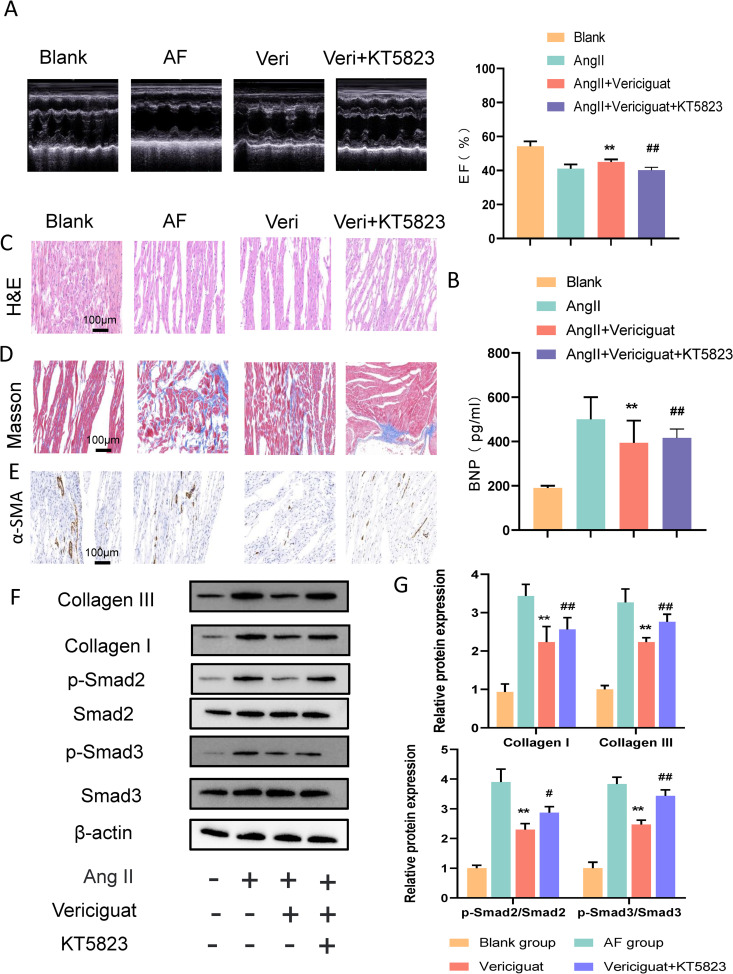
Vericiguat alleviates cardiac fibrosis in rats. (A) Echocardiographic measurements of mice hearts 10 weeks after Vericiguat or KT5823 for AF rats. Quantification of EF measurements in all groups (n = 8). (B)Detection of BNP by ELISA (n = 3). (C) Hematoxylin-eosin staining (n = 3). (D) Masson staining (n = 3). (E) Immunohistochemical detection of α-SMA (n = 3) of rats left ventricles. Scale bars, 100 µm. (F) Detection of myocardial fibrosis of key proteins expression under different treatments in CMs. (G) qRT-PCR detection of mRNA changes in key genes of the myocardial fibrosis Values represent the mean ± SEM. ^**^p < 0.01 vs AF group, ^#^p < 0.05 versus vericiguat group, ^##^p < 0.01 vs vericiguat group.

Furthermore, we investigate the mechanism by which vericiguat regulates myocardial fibrosis during AF pathogenesis through the TGF-β1/smad2/3 pathway. In vitro, fibroblasts were treated with Ang II. Immunofluorescence demonstrated a significant upregulation of TGF-β1 (Fig 4A), while Western blot analysis revealed that, in comparison to the untreated group, vericiguat treatment resulted in a significant decrease in Smad2/3 protein levels (Fig 4B) and corresponding changes in mRNA levels (p < 0.05), as well as a significant improvement in the fibrosis indicators collagen III and collagen I (Fig 5C, p < 0.05). Overall, the therapeutic mechanism of vericiguat may be to inhibit myocardial fibrosis by inhibiting the TGF-β1/Smad2/3 pathway. Moreover, when KT5823 was used to inhibit PKG, the effect of vericiguat was weakened, indicating that vericiguat exerts its anti-fibrotic effect through the TGFβ signaling pathway via cGMP. (Fig 4C and 4D).

### Vericiguat alleviates left atrial fibrosis in AF model rats via the PKG pathway

In order to study the effect of vericiguat on AF, a rat AF model was constructed and the therapeutic effect of vericiguat on the rat AF model was evaluated based on the duration of AF and the degree of left atrial fibrosis. The electrocardiogram (ECG) of the AF model rats exhibited the disappearance of the P-wave and the appearance of the F-wave, which is a typical electrocardiogram of AF (S Fig 2A). The blank group did not demonstrate any AF rhythm throughout the entire experimental period (S Fig 2B). This indicates the successful establishment of a rat AF model. One month later, it was observed that the AF group exhibited an increase in BNP, a reduction in ejection fraction, and severe HF. In comparison to the AF group, the BNP and the ejection fraction decreased (Fig 5A and 5B, p < 0.05), The AF group exhibited significant pathological changes, including disordered arrangement of myocardial cells, partial rupture of myocardial fibers, and interstitial fibrosis. In comparison, the myocardial cells in the treatment group with vericiguat exhibited a relatively orderly arrangement, a more complete and distinct structure, a notable reduction in inflammatory cell infiltration, and a significant alleviation of pathological changes (Fig 5C). The Masson staining results demonstrated that the degree of left atrial fibrosis in the AF group was significantly higher than that in the blank group. In contrast, the blue-stained collagen fibers in the myocardial tissue of the vericiguat treated group rats exhibited a significant reduction and were arranged in a neat and orderly manner (Fig 5D). Furthermore, immunofluorescence showed a significant decrease in α-SMA after vericiguat treatment compared with that in the AF group (Fig 5E). As demonstrated by Western blot analysis, vericiguat treatment led to a substantial reduction in Smad2/3 protein levels (Fig 5F) and the associated alterations in mRNA levels (p < 0.05). Additionally, a significant enhancement in fibrosis indicators collagen III and collagen I was observed (Fig 5G, p < 0.05). In summary, the therapeutic mechanism of vericiguat may be to inhibit myocardial fibrosis by regulating the TGF-β1/Smad2/3 pathway. The aforementioned results indicate that following intervention with vericiguat, there was a significant reduction in the degree of left atrial fibrosis. This finding demonstrates the efficacy of vericiguat in not only managing HF but also impeding the progression of myocardial fibrosis. This process depends on PKG.

## Discussion

AF and HF are two major diseases with high morbidity and mortality rates. They often have a complex etiology, with multiple risk factors, including age, hypertension, diabetes and structural heart disease, interacting in a way that is often associated with poor prognosis [21–23]. AF and HF can interact with each other through pathophysiological mechanisms, such as inducing cardiac remodeling [24]. On the one hand, the excessive activation of the neuroendocrine system caused by HF can promote the development and maintenance of AF. On the other hand, AF causes the heart to lose the atrial auxiliary pump function, affecting the heart’s pumping function, resulting in decreased cardiac output and stroke volume compared with sinus rhythm, which worsens cardiac function [22,25,26]. Some patients with AF and HF exhibit poor cardiac function and are unable to tolerate radiofrequency ablation [27]. In addition to basic anti-heart failure drugs, patients require drugs to control ventricular rhythm and anticoagulant drugs, which results in a higher number of drugs being taken and a poorer quality of life. Consequently, we hope to find a drug that can not only treat heart failure, but also control the recurrence of atrial fibrillation.

Vericiguat represents a novel therapeutic option for the treatment of Chronic HF [28]. As an sGC agonist, it can directly stimulate sGC through NO binding site-independent mechanisms, and play a synergistic role with endogenous NO to increase the sensitivity of sGC to NO and enhance the SGC-cyclic guanosine monophosphate signaling pathway. It can concentrate the double repair of NO-sGC- cGMP signaling pathways, promote small arterial vasodilation, accelerate vascular remodeling, reduce myocardial cell damage, thus inhibiting myocardial hypertrophy and ventricular remodeling, and improve the structure of the heart [29–31]. Furthermore, it can inhibit the oxidation of the body, alleviate the inflammatory response of myocardial cells, reduce the inflammatory damage of cells, inhibit cell apoptosis, improve the function of vascular endothelial cells, avoid myocardial thickening and ventricular remodeling, and thus improve cardiac function [32,33]. We demonstrated that NT-proBNP levels were lower and LVEF was higher in the treatment group following vericiguat treatment. These findings are consistent with those of previous studies [15,34]. Furthermore, the incidence of atrial fibrillation was notably diminished following treatment with vericiguat. In light of the findings of previous studies indicating that sGC agonists can mitigate renal and hepatic fibrosis to varying degrees [15], we conducted an investigation to ascertain whether vericiguat treatment could similarly reduce fibrosis indicators. Our findings revealed a notable decline in fibrosis indicators following vericiguat administration, suggesting a potential role for vericiguat in reducing the recurrence of atrial fibrillation by inhibiting myocardial fibrosis.

Myocardial fibrosis is a change in heart tissue that may lead to abnormal cardiac electrical signal transmission, thereby increasing the risk of the occurrence and recurrence of AF [35]. Previous studies have demonstrated that myocardial fibrosis is a significant contributor to AF, with the recurrence of AF being associated with the degree of myocardial fibrosis [36,37]. This is because myocardial fibrosis can alter the structure and function of the heart, making the electrical activity of the heart unstable [38,39]. These findings align with our clinical research results. The TGF-β1/Smad pathway is a critical mediator of myocardial fibrosis [27]. The results of our study indicate that vericiguat exerts an inhibitory effect on myocardial fibrosis by regulating the TGF-β1/Smad pathway. Subsequent investigations demonstrated that vericiguat modulates the TGF-β1/Smad pathway through the action of PKG, which may be attributable to the activation of the cGMP-PKG signaling pathway by stimulating sGC [40]. However, cGMP functions as an upstream regulator in the TGF-β1/Smad signaling pathway [41]. This study is the inaugural demonstration that vericiguat inhibits TGF-β1/Smad signaling through the PKG pathway, thereby reducing myocardial fibrosis and atrial fibrillation recurrence.

However, Ponikowski et al. conducted a specific investigation into the effect of vericiguat on AF, utilizing data from 5,050 patients in the VICTORIA study and conducting a retrospective analysis [42]. Their findings indicated that vericiguat did not affect the occurrence of AF. The following two reasons are taken into consideration: 1. Firstly, the race of the subjects in the study differed. Ponikowski’s study focused on patients from the United States, while our study concentrated on patients from Asia. 2. The sample size of our study was not sufficiently large. However, research has also demonstrated that Vericiguat exerts a positive effect on AF-related structural and electrical remodeling [43]. These findings suggest that Vericiguat may have therapeutic potential in the treatment of AF.

## Conclusion

In conclusion, vericiguat is not only efficacious in treating HF but also inhibits CFs to myofibroblasts, thereby reducing myocardial fibrosis and controlling the recurrence of AF. Nevertheless, it should be noted that this study has certain limitations.Nevertheless, given the limited sample size and the influence of patient ethnicity, future studies will include a larger sample size, data sharing with international teams, and analysis of interactions such as ethnicity and environment.

## Supporting information

S1 Fig Effects of vericiguat on cardiac cGMP/PKG signalling protein levels in rats with AngII-induced cardiomyopathy. (A) Representative images of western blotting. (B) Densitometry analyses of immunoreactive bands. **p < 0.01 versus Blank group, ^##^p < 0.01 versus AngII group).
(PDF)

S2 Fig Electrocardiograms of rats in different groups. (A) the control group. (B) the atrial fibrillation group.
(PDF)

S1 Table Primers used for plasmid construction and qRT-PCR. GAPDH, glyceraldehyde 3‑phosphate dehydrogenase.
(PDF)
