# Supplementary figures and images for: Vericiguat reduces atrial fibrillation recurrence by alleviating myocardial fibrosis via the TGF-β1/Smad2/3 pathway
Source: PLoS One. 2025 Jul 18;20(7):e0328272. doi: 10.1371/journal.pone.0328272 (PMC12274009; doi:10.1371/journal.pone.0328272)

A

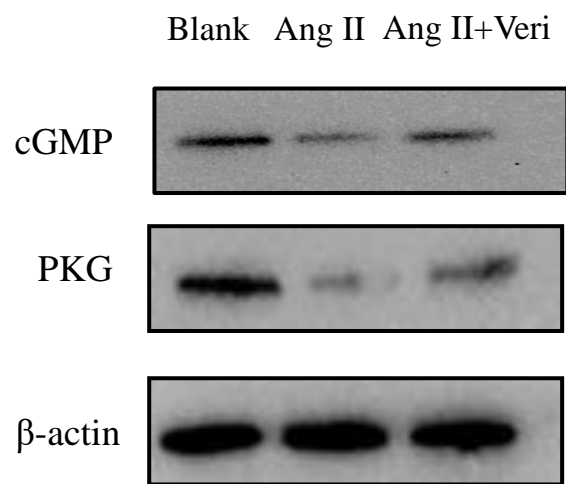

B

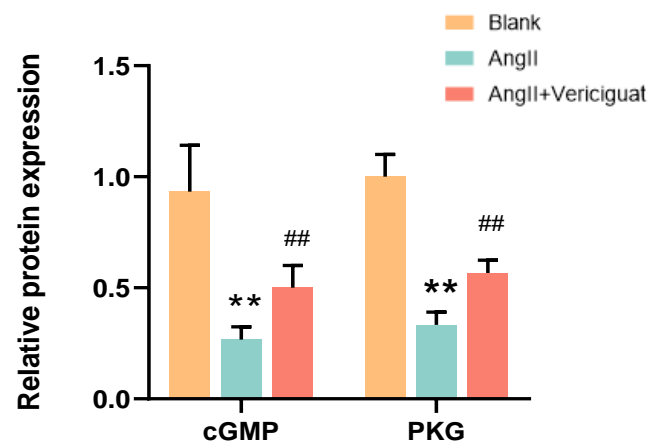

Supplement: S1 Fig — (A) Representative images of western blotting. (B) Densitometry analyses of immunoreactive bands. **p < 0.01 versus Blank group, ##p < 0.01 versus AngII group). (PDF) [file pone.0328272.s001.pdf]

A

AF

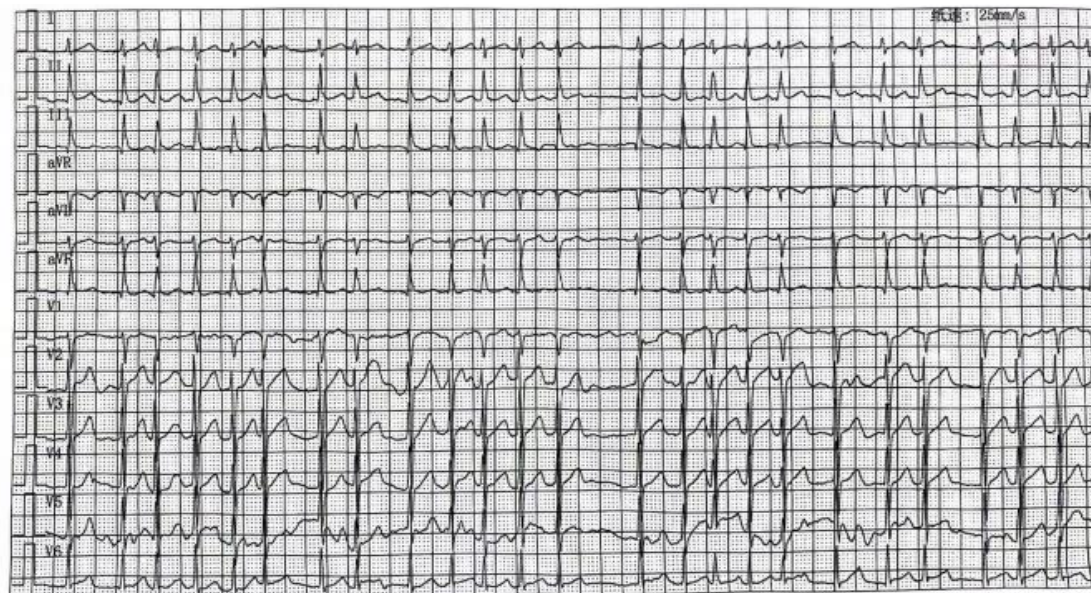

B

Control

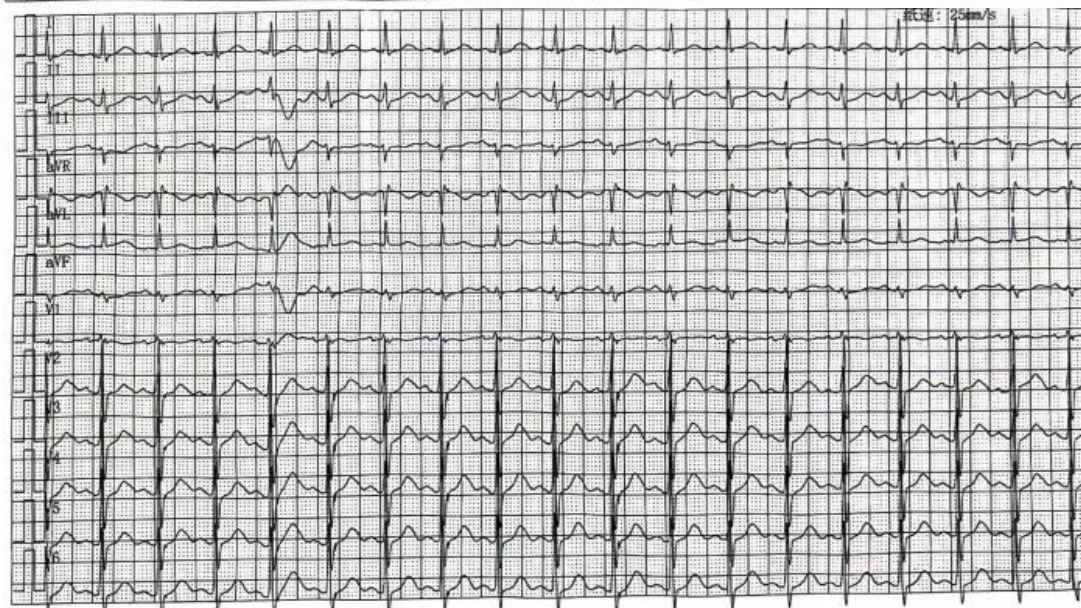

Supplement: S2 Fig — (A) the control group. (B) the atrial fibrillation group. (PDF) [file pone.0328272.s002.pdf]
