# Supplementary material for: Vericiguat reduces atrial fibrillation recurrence by alleviating myocardial fibrosis via the TGF-β1/Smad2/3 pathway
Source: PLoS One. 2025 Jul 18;20(7):e0328272. doi: 10.1371/journal.pone.0328272 (PMC12274009; doi:10.1371/journal.pone.0328272)
Supplement: S1 Table — GAPDH, glyceraldehyde 3‑phosphate dehydrogenase. (PDF) [file pone.0328272.s003.pdf]

Table 1. Primers used for plasmid construction and qRT-PCR

Gene	Primer sequence (5'-3')
α-SMA	F: ATGCAGAAGGAGATCACAGCTTTGG
	R:GTAGACAGGGAGGCGAGGATGG
collagen I	F:GACAGGCGAACAAGGTGACAGAG
	R: CAGGAGAACCAGGAGAACCAGGAG
collagen III	F: TGAAGGGCAGGGAACAACTTGATG
	R: GGATGAAGCAGAGCGAGAAGTAGC
fibronectin	F: AGGCACAAGGTCCGAGAAGAGG
	R: CATGAGTCATCCGTAGGCTGGTTC
TGF-β1	F: GGCCAGATCCTGTCCAAGC
	R: GGCCAGATCCTGTCCAAGC
GAPDH	F: GGAAAGCTGTGGCGTGATGG
	R: GTAGGCCATGAGGTCCACCA

GAPDH, glyceraldehyde 3-phosphate dehydrogenase.
